# HER-2 positive breast cancer is associated with an increased risk of positive cavity margins after initial lumpectomy

**DOI:** 10.1186/1477-7819-12-289

**Published:** 2014-09-20

**Authors:** Haixia Jia, Weijuan Jia, Yaping Yang, Shunrong Li, Huiyi Feng, Jieqiong Liu, Nanyan Rao, Liang Jin, Jiannan Wu, Ru Gu, Liling Zhu, Kai Chen, Heran Deng, Yunjie Zeng, Qiang Liu, Erwei Song, Fengxi Su

**Affiliations:** Department of Breast Surgery, Sun Yat-sen Memorial Hospital, Sun Yat-sen University, 107 Yanjiangxi Road, Guangzhou, 510120 PR China; Department of Pathology, Sun Yat-sen Memorial Hospital, Sun Yat-sen University, 107 Yanjiangxi Road, Guangzhou, 510120 PR China; Department of Breast Surgery, Second Affiliated Hospital of Guangzhou Medical University, 250 Changgang Road, Guangzhou, 510260 PR China; Department of Breast Surgery, Chancheng District Central Hospital, Sanyounan Road, Chancheng, Foshan 528031 PR China

**Keywords:** Breast cancer subtype, Breast-conserving surgery, Cavity margin, HER-2

## Abstract

**Background:**

The effect of breast cancer subtype on margin status after lumpectomy remains unclear. This study aims to determine whether approximated breast cancer subtype is associated with positive margins after lumpectomy, which could be used to determine if there is an increased risk of developing local recurrence (LR) following breast-conserving surgery.

**Methods:**

We studied 1,032 consecutive patients with invasive cancer who received lumpectomies and cavity margin (CM) assessments from January 2003 to November 2012. The following data were collected: patient age, cT stage, pT stage, grade, status of CM, lymph node status, menopausal status, ER, PR, HER-2, and Ki67, as well as the presence of extensive intraductal component (EIC) and lymphovascular invasion (LVI). A χ^2^ test was used to compare categorical baseline characteristics. Univariate and multivariate logistic regression analyses were performed to evaluate associations between pathologic features of CM status. Kaplan-Meier actuarial cumulative rates of LR (ipsilateral in-breast) were calculated.

**Results:**

A total of 7,884 pieces of marginal tissue were collected from 1,032 patients, and 209 patients had positive CMs. Of the patients tested, 52.3% had luminal A subtype, 14.9% were luminal B, 12.8% were luminal-HER-2, 8.1% were HER-2 enriched, and 11.8% were triple negative. Univariate analysis showed that EIC (*P* <0.001), LVI (*P* = 0.026), pN stage (N1 vs. N0: *P* = 0.018; N3 vs. N0: *P* <0.001), and luminal B (*P* = 0.001) and HER-2 (*P* <0.001) subtypes were associated with positive CMs. Multivariable analysis indicated that only EIC (*P* <0.001), pN stage (*P* = 0.003), and HER-2 subtype (*P* <0.001) were significantly correlated with positive CMs. On multivariable analysis, HER-2 subtype was an independent prognostic factor in LR (*P* = 0.031).

**Conclusions:**

The HER-2 subtype was the predictive factor most associated with positive CMs and an independent prognostic factor for LR. This result suggests that the increased risk of LR in HER-2 breast cancer is due to an increased microscopic invasive tumor burden, which is indicated by margin status after lumpectomy.

**Electronic supplementary material:**

The online version of this article (doi:10.1186/1477-7819-12-289) contains supplementary material, which is available to authorized users.

## Background

DNA microarray profiles have been used to classify breast tumors into distinct biologic subtypes [1, 2]. This testing may not often be feasible in a clinical setting, and these subtypes can be approximated by the expression of immunohistochemically-defined biological markers, such as the estrogen receptor (ER), the progesterone receptor (PR), and the human epidermal growth factor receptor 2 (HER-2), to classify tumors as luminal A (ER^+^ or PR^+^ and HER-2^−^), luminal B (ER^+^ or PR^+^ and HER-2^+^), HER-2^+^ (ER^−^ and PR^−^ and HER-2^+^), or triple-negative (TN) (ER^−^ and PR^−^ and HER-2^−^) subtypes [3]. Most reports show that the luminal A subtype is associated with the best prognosis, whereas significantly worse prognoses have been observed for the HER-2 and TN subgroups [4–6].

Randomized trials have validated breast-conserving surgery (BCS) as the standard treatment for early stage breast cancer (BC) [7, 8]. Minimizing local recurrence (LR) in the breast is very important in clinical settings because LR is associated with reduced survival and emotional distress [9]. The status of the surgical margin has been shown to be an independent predictor of LR [10, 11]. In a review including 34 related studies, LR was increased in cases involving a persistent, positive margin [12]. Negative margins should be achieved during BCS as recommended by the National Comprehensive Cancer Network guidelines.

Many studies have demonstrated that patients with HER-2 overexpression and TN BCs are at increased risk of developing LR following BCS [6, 13, 14]. Does the higher risk of LR in the two subtypes result from an increased microscopic invasive tumor burden that could be indicated by margin status after lumpectomy? We have no definite answer at present. Positive margins were reported to be significantly associated with large tumor size, young age, positive nodes, presence of lymphovascular invasion (LVI), and presence of an extensive intraductal component (EIC) [15–17]. In addition to the above analyzed clinical and pathologic variables, molecular phenotype may be a relevant factor of positive margins. The purpose of this study was to determine whether BC subtype approximation is associated with positive margins after initial lumpectomy and the extent of initial surgery that should be considered according to molecular subtypes.

## Methods

### Patient selection

We retrospectively reviewed the clinical and histopathologic data of 1,032 consecutive women ranging from 22 to 89 years (mean 48.2 years, median 47 years) at the time of diagnosis with clinical stage I or II invasive BC deemed suitable for BCS treated with lumpectomy and cavity margin (CM) excision between January 2003 and November 2012 in our center. The following data were included in the analysis: patient age, clinical T stage, pathological T stage, tumor grade, CM status, lymph nodes status, menopausal status, ER, PR, HER-2, and Ki67, as well as the presence of EIC and LVI (Table 1). Patients treated with prior neoadjuvant chemotherapy, endocrine therapy or radiation therapy to the breast or chest wall were excluded from the analysis, and patients with synchronous bilateral BC, more than one malignancy in one breast, or the presence of a non-palpable tumor were also excluded. We obtained approval for this study from the Institutional Review Board at Sun Yat-sen Memorial Hospital.

**Table 1:** **Patient baseline characteristics (n = 1,032)**

Characteristics	n	%
Age (y)		
≤35	110	10.7
36–50	527	51.1
>50	395	38.3
cT stage		
T1	575	55.7
T2	398	38.5
Tx	59	5.7
pT stage		
T1	658	63.8
T2	271	26.2
Tx	103	10.0
pN stage		
N0	736	71.3
N1	209	20.3
N2	50	4.8
N3	30	3.9
Unknown	7	0.7
Grade		
G1	83	8.1
G2	590	57.4
G3	354	34.5
Menopausal status		
Pre-	652	63.2
Post-	379	36.8
Margin status		
Negative	823	79.7
Carcinoma *in situ*	87	8.4
Invasive carcinoma	122	11.8
Presence of LVI	114	11.9
Presence of EIC	102	9.9
ER or PR positive	826	80.0
HER-2 positive	216	12.0
Histological subtype		
Invasive ductal carcinoma	805	78.0
Presence of DCIS component	83	8.0
Invasive lobular carcinoma	29	2.8
Other	115	11.2
Breast cancer subtype		
Luminal A	540	52.3
Luminal B	154	14.9
Luminal-HER-2	132	12.8
HER-2 positive	84	8.1
TN	122	11.8

EIC, Extensive intraductal component; LVI, Lymphovascular invasion; ER, Estrogen receptor; PR, Progesterone receptor; HER-2, Human epidermal growth factor receptor 2; DCIS, Ductal carcinoma in situ; IDC, Invasive ductal carcinoma; TN, Triple-negative.

### Surgical and pathological considerations

There are two primary approaches that are currently used for surgical margin assessment after initial lumpectomy: lumpectomy margin [18, 19] (LM, excision of the specimen containing the tumor) and CM (breast tissue sampled from resection bed cavity) [20, 21]. CMs are increasingly used for a comprehensive assessment of margin status, avoiding an unnecessary second operation in nearly half of patients to achieve negative margins and with the belief that these represent true margins and supersede LMs [20–23]. In our center, CM excision was employed as a routine part of lumpectomies. Physical examination, ultrasound, mammogram, and occasionally magnetic resonance imaging were used for BC diagnosis. For the lumpectomy, 1 cm of macroscopically normal tissue was removed to ensure that the margins of the removed specimens would be tumor free. The superficial and deep margins of the excision extended up to the skin and to the pectoralis fascia. Following excision of the main tumor, seven to nine rectangular CMs [length × width × thickness: (5–10 mm) × (5–10 mm) × (5 mm)] within the perimeter of the lumpectomy resection cavity were excised in a clockwise direction at the time of the lumpectomy, as reported previously [23]. After the resection of each CM, a silk suture was stitched to mark its location within the lumpectomy cavity, and CMs were submitted separately for histopathologic analysis. CMs were defined as positive when tumor cells were observed, regardless of whether they were carcinoma *in situ* or microscopic invasive carcinoma and independent of their distance from the true margin. Patients were recommended for further surgery for re-excision or mastectomy when one or more positive CMs were found. This procedure was well described in our prior study [24].

### Classification of subtypes

Breast cancers expressing high levels of Ki67 have been found to be associated with worse outcomes [25, 26]. In 2009, Cheang et al. [27] determined that the optimal cutoff point for the Ki67 labeling index was 13.25% for distinguishing luminal B from luminal A subtype. Based on this classification, the 12th St. Gallen International Breast Cancer Conference (2011) Expert Panel adopted a new immunohistochemical (IHC) classification of intrinsic subtypes, following application of the Ki67 labeling index using 14% as the cutoff value [28]. Therefore, biological cancer subtypes are approximated as follows: luminal A (ER^+^ or PR^+^ and HER-2^−^, Ki67 < 14%), luminal B (ER^+^ or PR^+^ and HER-2^−^, Ki67 ≥ 14%), luminal-HER-2 (ER^+^ or PR^+^ and HER-2^+^), HER-2 (ER^−^ and PR^−^ and HER-2^+^), and triple-negative (TN, ER^−^ and PR^−^ and HER-2^−^). This new classification was employed in our study. ER and PR statuses were determined using IHC staining. Positive ER or PR status was defined as ≥10% of tumor cell nuclei showing specific staining. An intensity of 0 to 1+ was considered HER-2 negative. Tumors were considered HER-2 positive if they were scored as 3+ by IHC or as 2+ by IHC and confirmed by fluorescence *in situ* hybridization amplification [29]. Grading of tumors was based on the modified Black’s nuclear grading system [30].

### Statistical analysis

A χ^2^ test was used to compare baseline characteristics among categorical variables. Margin status were analyzed by using univariate and multivariate logistic regression models. Associations with LR (ipsilateral in-breast) after BCS were evaluated using univariate and multivariate Cox proportional hazards regression models and summarized with hazard ratios and 95% confidence intervals (CIs). Kaplan-Meier actuarial cumulative rates of LR were calculated. All statistical tests were two-sided and considered statistically significant at 0.05. We performed all data analysis using SPSS 19.0 for Windows.

## Results

### Baseline characteristics stratified according to breast cancer (BC) subtype

There were significant differences between the five BC subtypes in the distribution of age (*P* = 0.030), histological subtype (*P* <0.001), cT stage (*P* = 0.034), pT stage (*P* = 0.006), grade (*P* <0.001), and EIC (*P* <0.001; Additional file 1: Table S1). In the study, we found that compared to the other BC subtypes, the TN subtype was most commonly observed at an age of 36 to 50 years, and the HER-2 subtype was commonly observed at an age of >50 years and frequently exhibited EIC, larger tumor size, and positive margins.

### Baseline characteristics stratified according to age quartile

There were significant differences among the three age quartiles in the distribution of BC subtype (*P* = 0.030), LVI (*P* = 0.006), and pT stage (*P* = 0.002; Additional file 2: Table S2). Compared to older patients, younger women more frequently had BC exhibiting LVI. To our surprise, in our study, older women more frequently had BC with larger tumors.

### Rate of positive cavity margins (CMs) by age quartile and breast cancer (BC) subtype

Table 2 provides an analysis of positive CMs by age quartile and BC subtype. We did not see any differences in positive CMs by BC subtype in the age group ≤35 years (*P* = 0.204). In contrast, there were significant differences in positive CMs between the two older age quartiles, middle age (*P* <0.001) and >50 years (*P* = 0.001). In the middle age quartile, 40.6% and 42.9% of patients with luminal B and HER-2 subtypes, respectively, had positive CMs, which was higher than in older age quartile. In the >50 years age group, only patients with HER-2 subtypes had higher CM positivity.

**Table 2:** **Rate of positive CMs by age quartile and BC subtype**

Age (years)	Luminal A n = 540 (%)	Luminal B n = 154 (%)	Luminal-HER-2 n = 132 (%)	HER-2 n = 83 (%)	TN n = 122 (%)	*P*value
≤35 (n = 110)	18.0	20.0	0.0	22.2	0.0	0.204
36–50 (n = 527)	17.8	40.6	24.7	42.9	12.2	<0.001
>50 (n = 395)	14.5	18.3	23.4	42.5	11.8	0.001

Positive CMs include ductal carcinoma *in situ* and invasive carcinoma.

### Univariate and multivariate analysis: clinicopathological features associated with positive margins

By univariate analysis, age, cT stage, menopausal status, and tumor grade were not statistically significantly correlated with positive CMs. However, the presence of EIC (OR = 2.77, 95% CI: 1.80–4.27, *P* <0.001), LVI (OR = 1.64, 95% CI: 1.06–2.55, *P* = 0.026), pN stage (N1 vs. N0: OR = 1.56, 95% CI: 1.08–2.25, *P* = 0.018; N3 vs. N0: OR = 6.15, 95% CI: 2.92–12.98, *P* <0.001), histological subtype (presence of ductal carcinoma *in situ* (DCIS) component vs. IDC: OR = 2.42, 95% CI: 1.50–3.90, *P* <0.001) and BC subtype (luminal B vs. luminal A: OR = 2.03, 95% CI: 1.34–3.08, *P* = 0.001; HER-2 vs. luminal A: OR = 3.45, 95% CI: 2.11–5.63, *P* <0.001, Table 3) had a statistically significant correlation with positive CMs.

**Table 3:** **Univariate analysis: clinicopathological features correlated with positive cavity margins**

Variable	n	OR (95% CI)	*P*value
BC subtype			
Luminal B vs. luminal A	154	2.03 (1.34–3.08)	0.001
Luminal-HER-2 vs. luminal A	132	1.43 (0.89–2.28)	0.139
HER-2 vs. luminal A	84	3.45 (2.11–5.63)	<0.001
Triple-negative vs. luminal A	122	0.60 (0.33–1.12)	0.110
Grade			
G2 vs. G1	590	0.98 (0.56–1.73)	0.947
G3 vs. G1	354	0.99 (0.55–1.79)	0.977
Histological subtype			
Presence of DCIS vs. IDC	83	2.42 (1.50–3.90)	<0.001
ILC vs. IDC	29	1.83 (0.82–4.09)	0.142
Others vs. IDC	115	0.39 (0.20–0.76)	0.006
Menopausal status			
Post- vs. pre-	379	0.98 (0.71–1.34)	0.894
cT stage			
T2/3 vs. T1	398	0.97 (0.71–1.34)	0.873
EIC	102	2.77 (1.80–4.27)	<0.001
LVI	114	1.64 (1.06–2.55)	0.026
Age			
~50 vs. ≤35	527	1.71 (0.97–3.02)	0.063
>50 vs. ≤35	395	1.35 (0.75–2.44)	0.311
pN stage			
N1 vs. N0	209	1.56 (1.08–2.25)	0.018
N2 vs. N0	50	1.03 (0.49–2.18)	0.932
N3 vs. N0	30	6.15 (2.92–12.98)	<0.001
pT stage			
T2 vs. T1	271	1.17 (0.82–1.66)	0.382

Positive CMs include DCIS and invasive carcinoma.

EIC, Extensive intraductal component; LVI, Lymphovascular invasion; ER, Estrogen receptor; PR, Progesterone receptor; HER-2, Human epidermal growth factor receptor 2; DCIS, Ductal carcinoma in situ; IDC, Invasive ductal carcinoma; ILC, Invasive lobular carcinoma; TN, Triple-negative.

Only significant variables in the univariate analysis were applied to the multivariate analysis with logistic regression model. By multivariate analysis, EIC (OR = 2.58, 95% CI: 1.54–4.32, *P* <0.001), pN stage (N3 vs. N0: OR = 3.92, 95% CI: 1.60–9.62, *P* = 0.003), and the HER-2 BC subtype (HER-2 vs. luminal A: OR = 2.60, 95% CI: 1.48–4.57, *P* <0.001; Table 4) were significantly correlated with positive CMs.

**Table 4:** **Multivariate analysis: clinicopathological features correlated with positive cavity margins**

Variable	OR	95% CI	*P*value
**A. Positive CMs including DCIS and invasive carcinoma**			
EIC	2.58	1.54–4.32	<0.001
Luminal B vs. luminal A	1.22	0.73–2.04	0.456
Luminal-HER-2 vs. luminal A	1.08	0.63–1.86	0.774
HER-2 vs. luminal A	2.60	1.48–4.57	<0.001
Triple-negative vs. luminal A	0.71	0.37–1.39	0.320
N1 vs. N0	1.43	0.92–2.20	0.109
N2 vs. N0	1.17	0.51–2.70	0.706
N3 vs. N0	*3.92*	1.60–9.62	0.003
**B. Positive CM including DCIS only**			
EIC	4.50	2.59–7.84	<0.001
Luminal B vs. luminal A	2.42	1.26–4.67	0.008
Luminal-HER-2 vs. luminal A	2.06	1.07–3.99	0.032
HER-2 vs. luminal A	5.58	2.89–10.77	<0.001
Triple-negative vs. luminal A	0.64	0.22–1.87	0.414
**C. Positive CM including invasive cancer only**			
Presence of DCIS component vs. IDC	2.19	1.04–4.63	0.040
ILC vs. IDC	2.82	1.10–7.23	0.031
Other vs. IDC	0.75	0.33–1.70	0.488
pN stage			
N1 vs. N0	2.15	1.30–3.53	0.003
N2 vs. N0	1.41	0.53–3.77	0.490
N3 vs. N0	8.42	3.54–20.05	<0.001

DCIS, Ductal carcinoma *in situ*; IDC, Invasive ductal carcinoma; EIC, Extensive intraductal component; ILC, Invasive lobular carcinoma.

### LR based on breast cancer (BC) subtype

**Figure 1:**
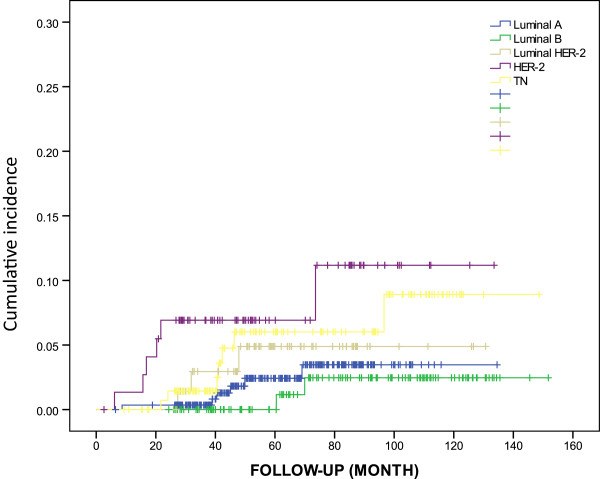
**Cumulative incidence of local recurrence (ipsilateral in breast) by breast cancer subtype.**

After a median follow-up of 63 months, a total of 831 patients who underwent successful conservative surgery with available follow-up records were reviewed for survival analysis. There were 32 LRs (ipsilateral in breast). The 5-year cumulative incidence of LR for all patients was 6.1% (95% CI: 3.3–8.9%). For patients in the luminal A subgroup, the 5-year cumulative incidence of LR was 3.9% (95% CI: 0.3–7.5%), compared with 4.4% (95% CI: 0–10.5%) for luminal B, 4.7% (95% CI: 0–10%) for luminal-HER-2, 13.4% (95% CI: 0–27.0%) for HER-2, and 8.8% (95% CI: 1.5–16.1%) for TN patients, respectively (Figure 1).

On univariable analysis, young age (36–50 vs. ≤35, *P* = 0.042), tumor size (T2 vs. T1, *P* = 0.014), positive nodes (LN^+^ vs. LN^−^, *P* = 0.032) and HER-2 subtype (HER-2 vs. luminal A, *P* = 0.016) were independently associated with increased risk of LR (Table 5). A multivariate Cox model revealed independent prognostic roles for tumor size (T2 vs. T1, *P* = 0.001), node status (LN^+^ vs. LN^−^, *P* = 0.044), and HER-2 subtype (HER-2 vs. luminal A, *P* = 0.031) in LR (Table 6).

**Table 5:** **Univariate survival analysis for LR (ipsilateral in breast)**

Variable	LR	
	HR (95% CI)	*P*
~50 vs. ≤35	0.33 (0.12–0.96)	0.042
≥50 vs. ≤35	0.78 (0.28–2.08)	0.589
T2 vs. T1	1.22 (1.04–1.44)	0.014
LN^+^ vs. LN^−^	2.41 (1.08–5.38)	0.032
G3 vs. G1	1.90 (0.95–3.80)	0.069
Luminal B vs. luminal A	0.59 (0.12–2.94)	0.517
Luminal-HER-2 vs. luminal A	1.86 (0.46–7.43)	0.381
HER-2 vs. luminal A	4.04 (1.30–12.54)	0.016
TN vs. luminal A	2.30 (0.77–6.87)	0.137

**Table 6:** **Multivariate survival analysis of LR (ipsilateral in breast)**

Variable	LR	
	HR (95% CI)	*P*
~50 vs. ≤35	0.384 (0.13–1.15)	0.086
≥50 vs. ≤35	1.031 (0.36–2.96)	0.955
T2 vs.T1	1.392 (1.15–1.69)	0.001
LN^+^ vs. LN^−^	2.348 (1.02–5.39)	0.044
Luminal B vs. luminal A	0.368 (0.07–1.99)	0.245
Luminal-HER-2 vs. luminal A	1.662 (0.40–6.84)	0.482
HER-2 vs. luminal A	3.650 (1.13–11.80)	0.031
TN vs. luminal A	2.025 (0.64–6.41)	0.230

## Discussion

In this study, we determined whether BC subtype, as approximated by ER, PR, HER-2, and Ki67, was associated with positive CMs of 1,032 consecutive women who underwent lumpectomies for early stage invasive BC. Compared to all other subtypes, the HER-2 positive subtype was an independent predictor of positive CMs (OR = 2.60, *P* <0.001) and an independent prognostic factor for LR (*P* = 0.016).

Many reports have shown that patients with the HER-2 subtype have an increased risk for LR after BCS and radiotherapy (RT) [6, 14]. In our study we found that HER-2 positive patients had a significantly higher recurrence risk, which is consistent with above studies. Randomized trials have demonstrated that the addition of trastuzumab to chemotherapy decreases LR by approximately 50% compared to treatment with chemotherapy alone [29]. The mechanisms underlying the high rate of LR in patients with the HER-2 subtype have not been conclusively determined. In a study, HER-2 status was reported to be the only primary tumor characteristic that correlated with the presence of circulating tumor cells [31]. Some groups have found that circulating tumor cells in operable BC patients are associated with worse prognosis [32]. In another study, patients with the HER-2 subtype were found to be more likely to have multicentric disease [33]. It was also reported that patients with the HER-2 subtype may be relatively resistant to post-lumpectomy RT [34, 35]. The above studies may partly explain the high rate of LR. Our finding that the HER-2 subtype was associated with an increase in positive CMs may lead to interpreting HER-2 BC with multicentric disease, which would result in increased residual microscopic tumors and higher LR to some extent. The follow-up results in our study showed that the HER-2 positive cancer had the highest LR, and maybe it is a reasonable verification of the above theory.

In a previous study, luminal BCs were reported to have a better prognosis [4, 5]. Interestingly, increased LR with the luminal B subtype among young women after BCS has been reported [35, 36]. In the current study, 40.6% of patients between the ages of 36 to 50 years with the luminal B subtype had positive CMs, which was higher than in the >50 years subgroup. Using univariate analysis with the luminal A subtype as the baseline, the luminal B subtype was associated with an increased rate of positive CMs, with an odds ratio of 2.03 (95% CI: 1.34–3.08, *P* = 0.001). This finding may partially explain the increased LR with the luminal B subtype. The mechanisms are still not well understood and need further study.

In our study, the TN subtype had a low rate of positive CMs, and the result did not seem to coincide with the higher LR of the TN subtype reported by most papers [4, 37, 38]. We observed the clinicopathologic features of the TN subgroup in the present study and found that this low rate of positive CMs may be related to the fact that most TN patients had T1 stage tumors (60.7%), less presence of LVI (TN vs. HER-2: 10.7% vs. 14.3%), and EIC (TN vs. HER-2: 4.1% vs. 22.6%). TN patients with large tumors may have received immediate mastectomies or neoadjuvant chemotherapy and would have thus been excluded from our study. This finding may reflect selection bias, but we have performed multivariate analyses to adjust for the confounding factors.

Our univariate analysis showed that the BC subtype, the presence of EIC or LVI, histopathology subtype, and pN stage were significantly associated with positive CMs. This result was not completely consistent with a previous study [20]. Cao et al. [20] reported that younger patient age, higher number of positive LMs, higher tumor grade, and the presence of EIC were predictive of residual carcinoma in CM specimens. In our study, age and high tumor grade were not predictive factors of positive CMs. Several previous studies have also demonstrated that the presence of EIC [17, 20] and larger tumor size [22, 39] were predictive factors for positive CMs. So far, we have only found one paper, reported by Sioshansi et al. [39], that was specifically looking for associations of different BC subtypes with the risk of residual tumors. Sioshansi et al. [39] showed that age (*P* = 0.003), tumor size (*P* <0.001), LVI (*P* = 0.007), nodal status (*P* <0.001), and TN subtype (*P* = 0.006) were associated with an elevated risk of residual invasive cancer by univariate analysis [39]. In our univariate analysis, EIC (*P* <0.001) was also an important predictive component of positive CMs, and this was not shown in the previous study. Using multivariable analysis, only nodal status (OR = 3.06, 95% CI: 1.77–5.30, *P* <0.001), TN status (TN vs. non-TN, OR = 3.28, 95% CI: 1.56–6.89, *P* = 0.02), and tumor size (tumor size >2.0 cm vs. <1.0 cm, OR = 3.49, 95% CI: 1.65–7.38, *P* = 0.001) maintained statistical significance on multivariate analysis [39]. However, tumor size was not a significant predictive factor associated with positive CMs in our multivariate analysis. EIC (OR = 2.58, 95% CI: 1.53–4.32, *P* <0.001), pN stage (N3 vs. N0: OR = 3.92, 95% CI: 1.60–9.62, *P* = 0.003), and HER-2 subtype (HER-2 vs. luminal A: OR = 2.60, 95% CI: 1.48–4.57, *P* <0.001) were significantly correlated with positive CMs. The difference between associated BC subtypes may be due to the following: i) Classification by different immunohistochemical markers. In previous studies, approximated molecular phenotypes were defined by ER, PR, and HER-2, which was different from our new classification. On the basis of recent data suggested by the 12th St. Gallen International Breast Cancer Conference (2011) Expert Panel, the Ki67 index was used in our study, which additionally discriminated partial luminal B patients from luminal A patients. The use of the Ki67 index is unique to this study. ii) Distribution of BC subtypes. Sioshansi et al. [39] reported that 73.5% of patients in their study were luminal A, 9.5% were luminal B, 4.5% were HER-2 enriched, and 12.5% were TN. In our study, 52.3% were luminal A, 14.9% were luminal B, 12.8% were luminal-HER-2, 8.0% were HER-2, and 11.8% were TN.

Among different age groups, positive rate of CMs in different molecular subtypes is not clear yet. We analyzed positive rates of CMs (including invasive cancer and carcinoma *in situ*) by age quartile and BC subtype in the current study. Women aged ≤35 years with BC are reported to have a poor prognosis and for most women, and menopause happens around age 50. According to this, we divided patients into three groups. In the youngest age quartile (≤35 years), the positive CM rate demonstrated no significant difference (*P* = 0.204). In contrast, the quartile containing ages 36 to 50 years had positive CM rates of 40.6% and 40.9% in luminal B and HER-2 subtypes, respectively (*P* <0.001), and the quartile with patients older than 50 years had a positive CM rate of 42.5% with the HER-2 subtype, which reached statistical significance (*P* = 0.001). Thus, younger age (≤35 years) was not a risk factor for positive CMs in our study.

The risk of residual disease, including carcinoma *in situ* and invasive cancer (residual disease, including carcinoma *in situ* alone, was excluded from one study [39]), after lumpectomy has been examined in many studies [24, 40]. In recent decades, positive re-excision rates from 17% to 39% have been reported [20, 41–43]. In our current series, 20.3% (209/1,032) of patients had positive CMs, including carcinoma *in situ* and invasive cancer. This result was similar to those of previously published literature. For a comprehensive assessment, we also evaluated the positive CM rate with carcinoma *in situ* or invasive cancer alone using multivariate analysis. The positive rates were 9.6% (87/910, CMs with carcinoma *in situ* alone) and 12.9% (122/945, CMs with invasive cancer alone). For patients with positive CMs, including carcinoma *in situ*, EIC (*P* <0.001) and BC subtypes (HER-2 vs. luminal A: *P* <0.001; luminal B vs. luminal A: *P* = 0.008; luminal-HER-2 vs. luminal A: *P* = 0.032, Table 4) showed a significant association with positive CMs. For patients with positive CMs, including invasive cancer alone, histological subtype (presence of DCIS component vs. IDC, *P* = 0.040; invasive lobular carcinoma vs. IDC, *P* = 0.031) and pN stage (N1 vs. N0: *P* = 0.003; N3 vs. N0: *P* <0.001, Table 4) showed statistical correlation with positive CMs. BC subtype was no longer a relevant factor, which was not consistent with Sioshansi et al. [39].

There are several inherent limitations to this study. i) Although many surgeons increasingly prefer to use CMs for margin assessment, without information from long-term clinical follow-up, it is not clear whether CMs or LMs are superior. We used only the CM method without corresponding LM section analysis. ii) This is a single-center study, and the population was not representative of Chinese or Asian demographics. iii) BC subtypes approximated according to ER, PR, HER-2, and Ki67 are only a substitute for genotype-based molecular BC subtypes. Further studies will be needed to confirm the findings based on these new definitions.

## Conclusions

In summary, although there are potential limitations to this study, the findings showed that that the poor prognosis of the HER-2 subtype is due to increased residual microscopic tumor burden after lumpectomy. More clinical trials will be required to confirm our conclusion. This information may help surgeons to choose the most appropriate surgical treatment for each patient. Further study and follow-up data are required to confirm the findings from our study. Oncoplastic breast surgery and an increased “boost” in radiotherapy may be good choices for patients with the HER-2 subtype to reduce the microscopic tumor burden and to improve prognosis and cosmetic results.

## Electronic supplementary material

Additional file 1: Table S1.: Patient baseline characteristics stratified by subtype. (DOCX 29 KB)

Additional file 2: Table S2.: Patient baseline characteristics stratified by age quartile. (DOCX 43 KB)

## Abbreviations

BC: Breast cancer
BCS: Breast-conserving surgery
CI: Confidence intervals
CM: Cavity margin
DCIS: Ductal carcinoma *in situ*
EIC: Extensive intraductal component
ER: Estrogen receptor
HER-2: Human epidermal growth factor receptor 2
IDC: Invasive ductal carcinoma
IHC: Immunohistochemistry
LM: Lumpectomy margin
LR: Local recurrence
LVI: Lymphovascular invasion
PR: Progesterone receptor
TN: Triple-negative.
