# Supplementary material for: HER-2 positive breast cancer is associated with an increased risk of positive cavity margins after initial lumpectomy
Source: World J Surg Oncol. 2014 Sep 20;12:289. doi: 10.1186/1477-7819-12-289 (PMC4190445; doi:10.1186/1477-7819-12-289)
Supplement: Supplementary file 1 — Additional file 1: Table S1.: Patient baseline characteristics stratified by subtype. (DOCX 29 KB) [file 12957_2013_1768_MOESM1_ESM.docx]

**Table S1（related to table 1）.** Patient baseline characteristics stratiﬁed by subtype

| Characteristic | Total *n*=1032  （%） | Luminal A  *n*=540  （%） | Luminal B  *n*=154  （%） | Luminal-HER2  *n*=132  （%） | HER2  *n*=83  （%） | TN  *n*=122  （%） | *P* value |
| --- | --- | --- | --- | --- | --- | --- | --- |
| Age |  |  |  |  |  |  | 0.030 |
| ≤35 | 10.7 | 9.3 | 16.2 | 9.1 | 10.7 | 11.5 |  |
| 36-50 | 51.1 | 51.1 | 44.8 | 55.3 | 41.7 | 60.7 |  |
| >50 | 38.3 | 39.6 | 39.0 | 35.6 | 47.6 | 27.9 |  |
| Menopausal status 0.153 | | | | | | | |
| Pre- | 63.2 | 62.4 | 63.0 | 65.2 | 54.2 | 71.3 |  |
| Post- | 36.8 | 37.6 | 37.0 | 34.8 | 45.8 | 28.7 |  |
| Histological subtype <0.001 | | | | | | | |
| IDC | 78 | 75 | 85.7 | 83.3 | 75.0 | 77.9 |  |
| Presence of DCIS component | 8.0 | 6.7 | 3.2 | 14.4 | 21.4 | 4.1 |  |
| ILC | 2.8 | 4.1 | 3.2 | 0.8 | 0.0 | 0.8 |  |
| Unknown | 11.1 | 14.3 | 7.8 | 1.5 | 3.6 | 17.2 |  |
| cT stage 0.034 | | | | | | | |
| T1 | 59.1 | 62.3 | 58.0 | 61.1 | 55.6 | 46.6 |  |
| T2 | 40.9 | 37.7 | 42.0 | 38.9 | 44.4 | 53.4 |  |
| Grade <0.001 | | | | | | | |
| G1 | 8.1 | 14.0 | 3.9 | 0.8 | 0.0 | 0.8 |  |
| G2 | 57.4 | 75.6 | 56.5 | 43.2 | 21.4 | 49.0 |  |
| G3 | 34.5 | 10.4 | 39.6 | 56.1 | 78.6 | 80.2 |  |
| Presence of EIC | 9.9 | 8.3 | 5.8 | 18.2 | 22.6 | 4.1 | <0.001 |
| Presence of LVI | 11.1 | 9.3 | 12.4 | 15.2 | 14.3 | 10.7 | 0.265 |
| Positive CMs | 20.3 | 16.5 | 28.6 | 22.0 | 40.5 | 10.7 | <0. 001 |
| pT stage 0.006 | | | | | | | |
| pT1 | 63.8 | 69.1 | 54.5 | 60.6 | 56.0 | 60.7 |  |
| pT2 | 26.3 | 21.1 | 31.2 | 31.1 | 35.7 | 31.1 |  |
| Unknown | 10.0 | 9.8 | 14.3 | 8.3 | 8.3 | 8.2 |  |
| pN stage 0.291 | | | | | | | |
| N0 | 71.8 | 72.4 | 68.4 | 66.7 | 72.6 | 78.5 |  |
| N1 | 20.4 | 21.1 | 23.0 | 24.2 | 14.3 | 14.0 |  |
| N2 | 4.9 | 4.7 | 4.6 | 4.5 | 8.3 | 4.1 |  |
| N3 | 2.9 | 1.9 | 3.9 | 4.5 | 4.8 | 3.3 |  |
| Abbreviations: EIC, extensive intraductal component; LVI, lymphovascular invasion; ER, estrogen receptor; PR, progesterone receptor; HER-2, human epidermal growth factor receptor 2; DCIS, ductal carcinoma in situ; IDC, invasive ductal carcinoma. TN, triple-negative. | | | | | | | |
