# Supplementary material for: HER-2 positive breast cancer is associated with an increased risk of positive cavity margins after initial lumpectomy
Source: World J Surg Oncol. 2014 Sep 20;12:289. doi: 10.1186/1477-7819-12-289 (PMC4190445; doi:10.1186/1477-7819-12-289)
Supplement: Supplementary file 2 — Additional file 2: Table S2.: Patient baseline characteristics stratified by age quartile. (DOCX 43 KB) [file 12957_2013_1768_MOESM2_ESM.docx]

**Table S2 （related to table 1）.** Patient baseline characteristics stratiﬁed by age quartile

| Characteristic | Total (*n*=1032)  % | Patients by Age Quartile (years) | | | *P* value |
| --- | --- | --- | --- | --- | --- |
|  |  | ≤35(*n*=110)  % | ~50(*n*=527)  % | >50(*n*=395)  % |  |
| cT stage |  |  |  |  | 0.597 |
| T1 | 59.1 | 53.5 | 59.2 | 57.9 |  |
| T2/T3 | 40.9 | 36.5 | 40.8 | 42.1 |  |
| Grade |  |  |  |  | 0.600 |
| G1 | 8.1 | 8.2 | 8.6 | 7.4 |  |
| G2 | 57.4 | 54.5 | 59.3 | 55.8 |  |
| G3 | 34.5 | 37.3 | 32.1 | 36.8 |  |
| Pathological subtype | | | | | 0.229 |
| IDC | 78.0 | 77.3 | 76.1 | 80.8 |  |
| Presence of DCIS component | 8.0 | 9.1 | 8.7 | 6.8 |  |
| ILC | 2.8 | 0 | 3.8 | 2.3 |  |
| Unknown | 11.1 | 13.6 | 11.4 | 10.4 |  |
| Presence of EIC | 9.9 | 10.0 | 11.6 | 7.6 | 0.134 |
| Presence of LVI | 11.1 | 18.2 | 12.0 | 7.8 | 0.006 |
| Positive CMs | 20.3 | 14.5 | 22.6 | 18.7 | 0.103 |
| pT stage |  |  |  |  | 0.002 |
| pT1 | 63.8 | 66.4 | 62.6 | 64.6 |  |
| pT2 | 26.3 | 15.5 | 26.9 | 28.4 |  |
| Unknown | 10.0 | 18.2 | 10.4 | 7.1 |  |
| pN stage |  |  |  |  | 0.904 |
| N0 | 71.8 | 71.8 | 71.6 | 72.1 | . |
| N1 | 20.4 | 19.1 | 20.8 | 20.2 |  |
| N2 | 4.9 | 6.4 | 5.2 | 4.1 |  |
| N3 | 2.9 | 2.7 | 2.5 | 3.6 |  |
| BC subtype |  |  |  |  | 0.030 |
| Luminal A | 52.3 | 45.5 | 52.4 | 54.2 |  |
| Luminal B | 14.9 | 22.7 | 13.1 | 15.2 |  |
| Luminal-HER-2 | 12.8 | 10.9 | 13.9 | 11.9 |  |
| HER-2 | 8.1 | 8.2 | 6.6 | 10.1 |  |
| TN | 11.8 | 12.7 | 14.0 | 8.6 |  |
| Abbreviations: EIC, extensive intraductal component; LVI, lymphovascular invasion; ER, estrogen receptor; PR, progesterone receptor; HER-2, human epidermal growth factor receptor 2; DCIS, ductal carcinoma in situ; IDC, invasive ductal carcinoma; TN, triple-negative. | | | | | |
